# Effect of Human Ovarian Tissue Vitrification/Warming on the Expression of Genes Related to Folliculogenesis

**DOI:** 10.7508/ibj.2015.04.005

**Published:** 2015-10

**Authors:** Zahra Shams Mofarahe, Marefat Ghaffari Novin, Mina Jafarabadi, Mojdeh Salehnia, Mohsen Noroozian, Nassim Ghorbanmehr

**Affiliations:** 1 *Dept. of Biology and Anatomical Sciences, School of Medicine, Shahid Beheshti University of Medical Sciences, Tehran, Iran; *; 2 *Reproductive Health Research Center, Tehran University of Medical Sciences, Tehran, Iran; *; 3 *Dept. of Anatomical Sciences, Tarbiat Modares University, Tehran, Iran; *; 4 *Biotechnology Group, Faculty of Biological Sciences, Alzahra University, Tehran , Iran*

**Keywords:** Vitrification, Gene expression, Humans

## Abstract

**Background:**

Ovarian tissue cryopreservation is an alternative strategy to preserve the fertility of women predicted to undergo premature ovarian failure. This study was designed to evaluate the expression of folliculogenesis-related genes, including factor in the germline alpha (*FIGLA*), growth differentiation factor-9 (*GDF-9*), follicle-stimulating hormone receptor (*FSHR*), and *KIT LIGAND* after vitrification/warming of human ovarian tissue.

**Methods:**

Human ovarian tissue samples were collected from five transsexual women. In the laboratory, the ovarian medullary part was removed by a surgical blade, and the cortical tissue was cut into small pieces. Some pieces were vitrified and warmed and the others were considered as non-vitrified group (control). Follicular normality was assessed with morphological observation by a light microscope, and the expression of *FIGLA, KIT LIGAND, GDF-9*,, and *FSHR *genes was examined using real-time RT-PCR in both the vitrified and non-vitrified groups.

**Results:**

Overall, 85% of the follicles preserved their normal morphologic feature after warming. The percentage of normal follicles and the expression of *FIGLA, KIT LIGAND, GDF-9*, and FSHR genes were similar in both vitrified and non-vitrified groups (*P* > 0.05).

**Conclusion:**

Vitrification/warming of human ovarian tissue had no remarkable effect on the expression of folliculogenesis-related genes.

## INTRODUCTION

Ovarian tissue cryopreservation is an alternative strategy to preserve the fertility of women predicted to undergo premature ovarian failure due to cancer treatment, genetic disorders, or other certain diseases [1, 2]. This approach has the advantage of restoring both fertility and endocrine function [3] and may be the only acceptable method to preserve fertility for pre-pubertal girls [4], for women who cannot delay the start of cancer treatment and for women with hormone-sensitive malignancies [5].

There are two methods for cryopreservation of ovarian tissue: slow freezing and vitrification. Slow freezing, which was introduced earlier, is still available in clinical practice [6]; however, due to simplicity, safety, and inexpensiveness of vitrification technique, more attention has been recently given to this method [7].

Different studies have been conducted on the vitrification of human ovarian tissue and some of them have demonstrated the incidence of damage to the follicles [8-11]. Most studies, on the other hand, have reported that the majority of follicles in ovarian cortical tissue maintain their normal morphology and fine structure at the electron microscopy level after vitrification and warming [12-15].

The normal growth and development of follicles require the expression of specific genes involved in the folliculogenesis [16]. Factor in the germline alpha (*FIGLA*) gene plays an important role in the early stages of follicular development. It is exclusively expressed in primordial follicles and has a regulatory role in the expression of the zona pellucida genes and also in the formation of primordial follicles in the early events of folliculogenesis [17]. The mutation of this gene can cause early menopause [18]. In the later stages of development, growth differentiation factor-9 (*GDF-9*) gene is expressed in the primary follicle oocytes. It has been proposed to be an important regulator of early follicular growth, supporting oocyte viability [19], recruitment of theca cells [20], and finally, transition of primary follicles to secondary follicles [19]. 

Some developmental genes are expressed in granulosa cells, including *KIT LIGAND *gene that is expressed in the granulosa cells of primordial follicles and causes the follicular transition from primordial to primary follicles [21]. Follicle-stimulating hormone receptor (*FSHR*) is another gene that is expressed in the granulosa cells of secondary follicles, and causes follicular transition from secondary to antral follicles [22].

There is a dearth of report regarding the expression of some genes after vitrification/warming of the ovarian tissue [23, 24]. Recently, Abdollahi* et al.* [23] has shown that the expression of some apoptosis-related genes is changed and some of them are unchanged after vitrification/warming of the ovarian tissue. 

According to our knowledge, there is a lack of information on the expression of folliculogenesis-related genes involved in early follicular development after cryopreservation of human ovarian tissue. Thus, this study was designed to evaluate the expression of folliculogenesis-related genes, including *FIGLA, KIT LIGAND, GDF-9*, and *FSHR *after vitrification/ warming of human ovarian tissue.

## MATERIALS AND METHODS

All reagents and materials were obtained from Sigma-Alderich (Germany) except mentioned otherwise.

***Ovarian tissue collection. ***The ovarian tissue samples were collected from five transsexual women (female to male) aged 20-30 years old, suffering from gender identity disorder. The women were undergo sex reassignment surgery by hysterectomy and oophorectomy, under a protocol approved by the Ethics Committee of the Faculty of Medical Science of Shahid Beheshti University, Tehran, Iran (Ref. No.71). Then they were immediately transferred to the laboratory in pre-warmed and equilibrated Leibovitz'sL -15 medium supplemented with 10 mg/ml human serum albumin, 100 IU/ml penicillin, and 100 μg/ml streptomycin. Next, the ovarian medullary part was removed by a surgical blade, and the cortical tissue was cut into small pieces (approximately 2.5 × 1 × 1 mm) under a sterile condition. Finally, these fragments were randomly divided into vitrified and non-vitrified groups.

***Vitrification and warming procedure. ***The tissue samples were vitrified according to the method described earlier by Kagawa *et al. *(2009) with some modifications [12]. Briefly, the ovarian tissue samples were first rinsed in Hanks' balanced salt solution (HBSS) supplemented with 20% human serum albumin, and then equilibrated in HBSS containing 7.5% ethylene glycol (EG) and 7.5% dimethyl sulphoxide (DMSO) for 25 min. Next, they were transferred into the vitrification solution (20% EG, 20% DMSO, and 0.5 mol/l sucrose) for 15 min. Finally, the tissue samples were individually transferred into aseptic cryovials containing 100 μl vitrification solution, placed on nitrogen vapor for 30 s and then immersed and stored in liquid nitrogen for one week. 

The samples were warmed by immersing the vials in 37°C water bath with gentle agitation until defrosted. Next, they were transferred quickly into 1 mol/l sucrose in HBSS at 37°C for 3 min and then were moved into 0.5 mol/l sucrose at room temperature for 5 min. Finally, the samples were equilibrated in McCoy’s medium before any assessments.

***Histological evaluation by hematoxylin and eosin staining. ***Three fragments from five different ovaries in vitrified and non-vitrified groups (15 fragments in each group) were fixed in Bouin's solution for 12 h. They were subsequently embedded in paraffin wax (routine protocol) and serially sectioned at 5μm thickness. Every 10th section of each fragment was mounted on glass slides and stained with hematoxylin and eosin. Then each section was examined by a light microscope to count follicles (×10 objective). To avoid counting the follicles more than once, only those with a visible nucleus of oocytes were counted. The follicles were classified as primordial, primary, secondary, and antral according to Lass *et al.* [25]. Primordial follicles contained one layer of flattened granulosa cells; primary follicles had one layer of cuboidal granulosa cells; secondary follicles had two or more layers of cuboidal granulosa cells; and antral follicles showed multiple layers of cuboidal granulose cells with antrum. Normal follicles contained round oocytes, surrounded by granulosa cells in a close contact to each other. The ooplasm was homogenous with finely granulated nucleus. Atretic follicles had pyknotic oocyte nucleus, shrunken ooplasm or disorganized granulosa cells. 

***RNA extraction and cDNA synthesis for molecular assessment. ***In vitrified and non-vitrified groups (at least 9 fragments in each group), three to five fragments from three different ovarian samples were stored at -80ºC for subsequent molecular assessment. Total RNA was extracted from the vitrified and non-vitrified ovarian tissues using TRIzol reagent (Invitrogen, USA) according to the manufacturer’s instructions. The RNA samples were treated with DNase to eliminate any genomic DNA contamination just prior to proceed with the cDNA synthesis. Then the RNA concentration was determined by spectrophotometry and adjusted to a concentration of 250 ng/μl. Finally, 1000 ng of the extracted RNA was used for cDNA synthesis using the commercial Kit (Thermo Scientific, EU) according to the manufacturer’s instructions. Using oligodT, the extracted RNA was reverse transcribed by Moloney murine leukemia virus reverse transcriptase. The cDNA-synthesis reaction was performed at 42°C for 60 min, and the obtained cDNA was stored at -20ºC until use.

***Real-time RT-PCR. ***The primers for real-time RT-PCR were newly designed using the Gen Bank (http://www.ncbi.nlm.nih.gov) and online software such as Primer3 and Oligo Analyzer. As shown in Table 1, newly designed primers were ordered and synthesized at Generary Biotech Co. (China). One-step RT-PCR was performed using the Applied Biosystems (UK) real-time thermal cycler according to QuantiTect SYBR Green RT-PCR Kit (Applied Biosystems, UK, Lot no: 1201416). The housekeeping gene, *β-ACTIN*, was used as an internal control. One microliter of cDNA, 1 μl of the mixture of forward and reverse primers, and 10 μl of SYBR Green Master Mix were used per 20 μl of the reaction volume. After completing the PCR run, melt curve analysis was applied to confirm the amplified product and record the Ct values. A single, sudden decline of fluorescence during a melt curve at a high temperature indirectly indicates a specific amplicon being amplified during the PCR run; otherwise cDNA would be omitted and the cDNA of another fragment would be replaced. For each sample, the reference gene (*β-ACTIN*) and the target genes were amplified in the same run.

Real-time thermal condition included holding step at 95ºC for 5 min, cycling step at 95ºC for 15 s, 58ºC for 30 s, and 72ºC for 15 s, which was continued by a melt curve step at 95ºC for 15 s, 60ºC for 1 min, and 95ºC for 15 s. Then the relative quantification of target genes was determined using the Pfaffl method [26]. The real-time RT-PCR experiments were done in duplicate for each sample.

***Statistical analysis. ***Statistical analysis was carried out with the SPSS 19.0 software. Quantitative variables were expressed as mean ± SE and percentage. The normality of data was assessed by Kolmogrov-Smirnov test, and the homogeneity of variance was assessed by Levene's test. The results of real-time RT-PCR were compared by independent samples *t*-test. *P* values less than 0.05 were considered as statistically significant.

## RESULTS

**Fig.1:**
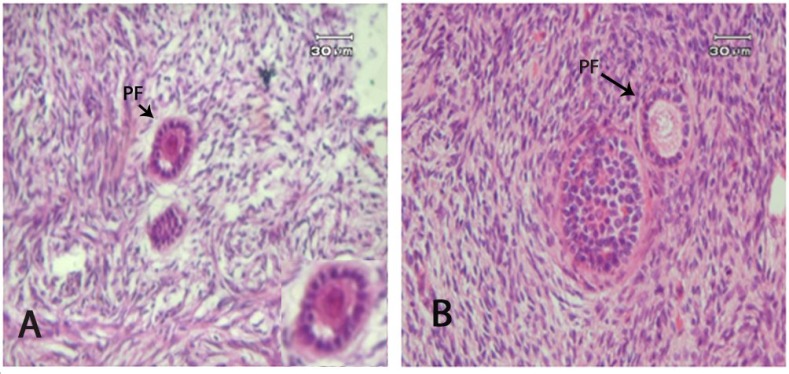
The staining of human ovarian tissue. The Figure shows morphology of primary follicle (PF) within human ovarian tissue in vitrified (A) and non-vitrified (B) tissue samples. The follicular integrity and stromal tissue structures were preserved. The detachment between oocyte and granulosa cells was observed

***Histological observation of ovarian tissue. ***The morphology of ovarian cortical sections in both the vitrified and non-vitrified groups is shown in Figure 1. The follicle contained round oocyte, surrounded by granulosa cells in close contact to each other. Stromal cells had spindle shaped nucleus with a finely diffused chromatin. After vitrification/warming, the follicular integrity and stromal tissue structures were preserved, however, detachment between oocyte and granulose cells were observed in some follicles (Fig. 1). No antral follicles were observed in any of the two groups.

***Follicular counting of ovarian tissue. ***A total of 510 follicles were counted and analyzed in both the vitrified and non-vitrified tissues (200 follicles in the vitrified and 310 follicles in the non-vitrified tissues). The percentages of the morphologically normal follicles at different developmental stages in both groups are summarized in Table 2. Overall, 85% of the follicles preserved normal morphologic feature and 15% were degenerated after warming. Among normal follicles, the proportion of primordial, primary, and secondary follicles was 57.7%, 25.2%, and 2.1%, respectively. There was no significant difference in the percentage of normal follicles at different developmental stages between the two groups (*P* > 0.05).

**Table 1:** The characteristic of primers used in real-time RT-PCR assays

**Accession number**	**Target gene**	**Primer sequence**	**Product size (bp)**
NM_001101.3	*β-ACTIN*	Forward: 5' –TCAGAGCAAGAGAGGCATCC- 3' Reverse: 5' –GGTCATCTTCTCACGGTTGG- 3'	187
NM_001004311.3	*FIGLA*	Forward: 5' –TCGTCCACTGAAAACCTCCAG- 3' Reverse: 5' –TTCTTATCCGCTCACGCTCC- 3'	76
NM_000899.4	*KIT LIGAND*	Forward: 5' –AATCCTCTCGTCAAAACTGAAGG- 3' Reverse: 5' –CCATCTCGCTTATCCAACACTGA- 3'	163
NM_005260	*GDF-9*	Forward: 5' –TCCACCCACACACCTGAAAT- 3' Reverse: 5' –GCAGCAAAACCAAAGGAGGA- 3'	147
NM_181446.2	*FSHR*	Forward: 5' –CTGGCAGAAGAGAATGAGTCC- 3' Reverse: 5' –TGAGGATGTTGTACCCGATGATA- 3'	157

**Fig. 2:**
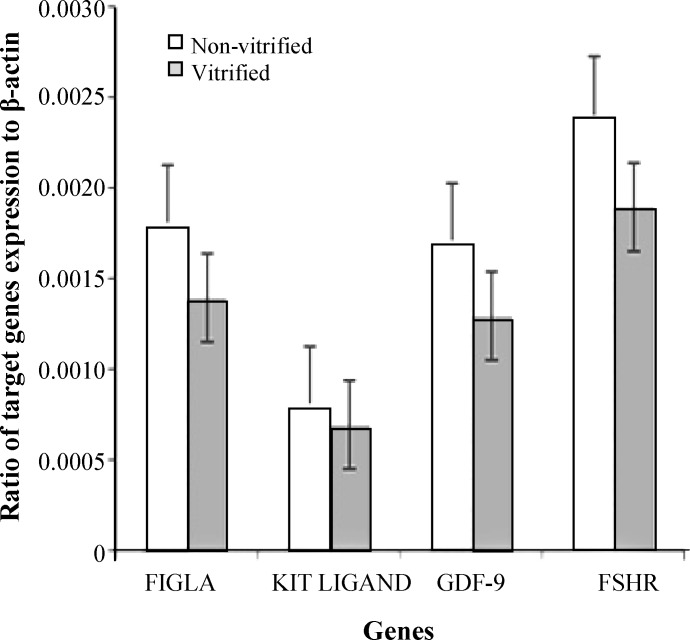
The ratio of folliculogenesis-related genes expression to *β-ACTIN*. The ratio of genes expression of *FIGLA, KIT LIGAND , GDF-9 *, and *FSHR * to *β-ACTIN* using real-time RT-PCR in vitrified and non-vitrified human ovarian tissues. Values are means ± SE. There is no statistically significant difference between the two groups (*P* > 0.05).

***Expression of folliculogenesis-related genes in ovarian tissue. ***The expression ratio of several target genes (*FIGLA, KIT LIGAND, GDF-9, *and *FSHR*) to housekeeping gene (*β-ACTIN*) in the vitrified and non-vitrified tissues is shown in Figure 2. The expression level of*FIGLA, KIT LIGAND, GDF-9*, and *FSHR *to *β-ACTIN* in the vitrified tissues were 14 ×10^-4^ ± 0.9 × 10^-4^, 7.3 × 10^-4 ^± 3.9 × 10^-4^, 13 × 10^-4 ^± 1.8 × 10^-4^, and 18.7 × 10^-4^ ± 0.3 × 10^-4^, respectively. This ratio in the non-vitrified tissues were 18.4 × 10^-4^ ± 7.7 × 10^-4^, 8.6 × 10^-4 ^± 2.9 × 10^-4^, 17.3 × 10^-4 ^± 5.6 × 10^-4^, and 24 × 10^-4 ^± 9.5 × 10^-4^, respectively. There was no significant difference between the target genes expression in the vitrified and non-vitrified tissues (*P* > 0.05). 

## DISCUSSION

Morphological observations in the present study showed that the majority of follicles in human ovarian cortical tissue maintained their normal structure after vitrification/warming. There was no significant difference in the percentage of intact follicles in the vitrified and non-vitrified tissues (*P* > 0.05). In addition, the integrity of ovarian stromal tissue was well preserved; therefore, it seems that this procedure of vitrification can be a good alternative for human ovarian tissue cryopreservation. The competence of this method may be the result of using EG, DMSO, and sucrose as cryoprotectants. EG has low toxic effect, rapid cell diffusion, the high compatibility with other cryoprotectants, and the best survival rate of follicle [27]. Most protocols yielding successful results use solutions containing a mixture of DMSO and EG [12, 13, 15, 28, 29]. This cryoprotectant compound has also been effective in preserving the ovarian tissue integrity of other species [8, 12]. Moreover, adding sucrose assists dehydration, decreases the risk of intracellular crystallization and reduces the toxic effects of permeable cryoprotectant in the vitrification solution [27]. The other reason for the competence of this method may be due to a large number of primordial follicles compared with other follicles in the ovarian cortical tissue [30]. Since primordial follicles contain immature dormant small oocytes, which are lack of zona and cortical granules; hence, they are more resistant to cryoinjury [31]. 

**Table 2:** The percentages of the normal follicles at different developmental stages in the human ovarian tissue samples

**Group**	**Total no. of follicles**	**No. of normal follicles (%mean ± SE)**	**No. of primordial follicles (%mean ± SE)**	**No. of primary ** **follicles (%mean ± SE)**	**No. of secondary follicles (%mean ± SE)**
Vitrified	200	171 (85.0 ± 1.3)	115 (57.7 ± 3.4)	52 (25.2 ± 3.0)	4 (2.1 ± 0.2 )
					
Non-vitrified	310	284 (90.4 ± 2.0)	199 (60.7 ± 7.1)	72 (25.0 ± 4.9)	13 (4.7 ± 1.8)

There is no significant difference in the percentage of normal follicles at different developmental stages between the two groups (*P* > 0.05).

There are few reports in the literature regarding the changes in the follicular and oocyte genes expression after vitrification/warming of human ovarian tissue [23]. This is the first attempt to investigate the folliculogenesis-related genes expression at molecular level.

In this study, the expression of *FIGLA *and* KIT LIGAND *genes related to primordial follicles as well as *GDF-9 * and *FSHR *genes corresponding to primary and secondary follicles were evaluated, and the results showed that the target genes expression was similar in the two study groups (*P* > 0.05). It appears that vitrification/warming using DMSO, EG, and sucrose as cryoprotectants has no remarkable effect on the expression of developmental genes related to primordial, primary, and secondary follicles. Proper expression of these genes is essential for follicular transition to the next stage of follicular development [32]. In agreement with our results, Herraiz *et al. *[1] have demonstrated that vitrification using EG, DMSO, and sucrose offer similar conditions to fresh tissue for *GAPDH *gene expression in bovine ovarian tissue. 

Few studies have been conducted regarding alteration in gene expression patterns after vitrification of ovarian tissue in other species [1, 24]. In contrast to our results, Choi *et al.* [24] have shown that angiogenic gene expression is decreased significantly in mouse ovarian tissue after vitrification/warming. Also, the expression of *GAPDH *gene in bovine ovarian tissue was diminished after vitrification with ethyl vinyl acetate bag [1]. Abdollahi* et al.* [23] reported that the expression of some apoptosis-related genes was changed and some intact. This discrepancy between these results can be due to differences in the methods of vitrification or different subjects of species.

Therefore, to better understand the effect of vitrification/warming on the gene expression of human ovarian tissue, more additional studies after long term *in vitro* culture or xenograft transplantation of the vitrified tissues are needed. 

In conclusion, our results, for the first time, demonstrated that in spite of some alterations in morphology of human ovarian tissue after vitrification using DMSO, EG, and sucrose, no remarkable effect was observed on the expression of folliculogenesis-related genes immediately after warming.
